# Photocatalytic Reduction of CO_2_ by ZnO Micro/nanomaterials with Different Morphologies and Ratios of {0001} Facets

**DOI:** 10.1038/srep38474

**Published:** 2016-12-06

**Authors:** Xiaodi Liu, Liqun Ye, Shanshan Liu, Yinping Li, Xiaoxu Ji

**Affiliations:** 1 College of Chemistry and Pharmaceutical Engineering, Nanyang Normal University, Nanyang, Henan, China; 2 College of Physics and Electronic Engineering, Nanyang Normal University, Nanyang, Henan 473061, China

## Abstract

ZnO microspheres, ZnO microflowers and ZnO nanorods are successfully synthesized *via* a convenient solvothermal method in distilled water-ethanol mixed medium. The as-prepared ZnO micro/nanomaterials are characterized by XRD, SEM, TEM, HRTEM, XPS, BET, and UV-Vis. The morphologies and exposed facets of the ZnO micro/nanomaterials can be controlled by simply changing the volume ratio of distilled water to ethanol, and their formation mechanisms are also proposed. In addition, the photocatalytic activities of the ZnO samples are investigated towards the photoreduction of CO_2_ to CO. It is found that ZnO nanorods with high ratio of {0001} facets and large surface areas possess higher CO formation rate (3.814 μmol g^−1^ h^−1^) in comparison with ZnO microspheres and ZnO microflowers (3.357 and 1.627 μmol g^−1^ h^−1^, respectively). The results can not only provide an important indication about the influence of the {0001} facets on the activity of CO_2_ photoreduction over ZnO, but also demonstrate a strategy for tuning the CO_2_ photoreduction performance by tailoring the surface structures of ZnO micro/nanomaterials.

Recently, the ever-increasing emission of CO_2_ in the atmosphere and the diminishing of fossil resources have driven researchers to control the atmospheric level of CO_2_ and explore renewable energy sources^1^. It is found that CO_2_ can be converted into energetic carbon fuels (*e.g.*, CO and CH_3_OH) with the assistant of solar energy and semiconductor photocatalysts, which is a promising and economical strategy^2^,^3^. The photocatalytic reaction usually occurs at the interface between the reactant (i.e., CO_2_) and the photocatalyst; therefore, the photocatalytic activity of the catalyst not only depends on the morphology but also is strictly related to the surface atomic and electronic structures^4^,^5^. What is more, it is well-known that different crystal facets of a semiconductor have different atomic arrangements and electronic structures, endowing them with distinctive photocatalytic activities^6^. Thus, crystal facet engineering is an exciting direction to pursue for highly active semiconducting photocatalysts, and increasing attention has been paid to the design and facet-controlled synthesis of catalysts to improve their activities for the photoreduction of CO_2_^7^,^8^,^9^,^10^,^11^. For example, Cheng’s group have reported that A-TiO_2_ nanorods with dominant (010) facet exhibit superior photocatalytic conversion of CO_2_ into CH_4_ than P25, which may be caused by the high conduction band (CB) edge of the TiO_2_ nanorods terminated with (010) facet and the stronger interaction of CO_2_ on the (010) facet than on the (001) and (101) facets^7^. Xie *et al*. have confirmed that the (002) facet makes the rectangular sheet-like WO_3_ have an elevated CB minimum, which is higher than the redox potential of CH_4_/CO_2_, so the photoreduction of CO_2_ by the electrons from WO_3_ can be occurred^8^. Nevertheless, in spite of the above successes, intensive investigations into this strategy are imperative, and it still remains a great challenge to research the influences of crystal facet on the activities of other photocatalysts for the CO_2_ conversion.

ZnO, with a wide direct band gap (3.37 eV) and a large exciton binding energy (60 meV), is a promising photocatalyst due to its high photosensitivity, environmental sustainability, thermal stability, and low-cost^12^. ZnO is considered as a suitable alternative to TiO_2_; however, only a few ZnO nanomaterials and ZnO-based composites have been employed in the photoredox CO_2_ conversion reactions^13^,^14^,^15^. Moreover, wurtize ZnO, with several alternating Zn-polar (0001) and O-polar (000-1) facets, is a typical polar crystal, and many extensive studies have indicated that ZnO with large ratio of {0001} planes exhibit enhanced photocatalytic performance, such as the degradation of organic pollutants^5^,^16^,^17^,^18^,^19^. However, such studies on the photocatalytic reduction of CO_2_ are limited. Hence, there is still much room for the development of ZnO with controlled exposed facets; furthermore, the understanding of the surface structure-performance relationship of ZnO may offer new opportunity for the construction of ZnO micro/nanomaterials with high activity for the CO_2_ photoreduction.

In this paper, we report the controlled synthesis of ZnO microsphere (S-1), ZnO microflowers (S-2), and ZnO nanorods (S-3) with different ratios of {0001} facets by a facile and efficient solvothermal method. The process is carried out in a series of distilled water-ethanol mixed solvent with different volume ratio of water to ethanol (*R*_w/e_). Among the three samples, S-3 exhibits the best activity for CO_2_ photoreduction under sunlight irradiation.

## Results

### Characterizations of S-1, S-2, and S-3

**Figure 1:**
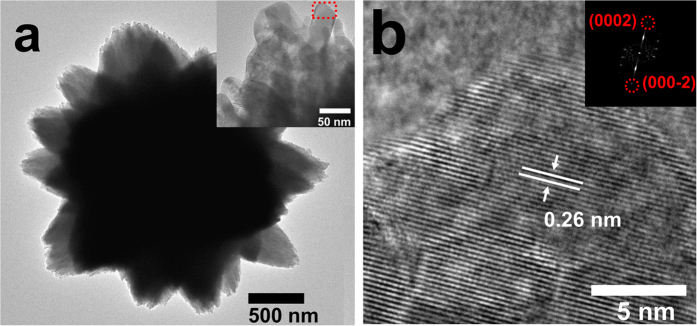
(**a**) TEM image of S-1, the inset is an enlarged TEM image of the edge of the microsphere; (**b**) HRTEM image of one ZnO nanocrystal originating from the red square of a, the inset is the corresponding FFT pattern along the [10-10] axis.

The morphologies and crystalline natures of the products are characterized by SEM, TEM, and HRTEM. SEM image (Fig. S1a, Supplementary Information) reveals that S-1 is consisted of urchin-like microspheres and the sizes of these microspheres are in the range of 1.5-5.0 μm. In fact, as displayed in the TEM image taken from a single sphere (Fig. 1a), the microsphere is composed of rod-like ZnO nanocrystals with an average diameter of ~30 nm. Figure 1b is the HRTEM image of a primary crystal originating from the red square in Fig. 1a. The clear lattice fringe with an interplanar distance of ~0.26 nm can be assigned to the (0002) atomic plane of hexagonal ZnO. The corresponding fast Fourier transform (FFT) pattern (inset of Fig. 1b, viewed from the [10-10] axis) has highly symmetrical dotted lattice, revealing the single-crystalline nature of the original nanocrystal; moreover, all the diffraction dots can be perfectly attributed to hexagonal ZnO. Both HRTEM image and FFT pattern demonstrate that the ZnO nanocrystals have a preferential growth orientation along the [0001] direction.

**Figure 2:**
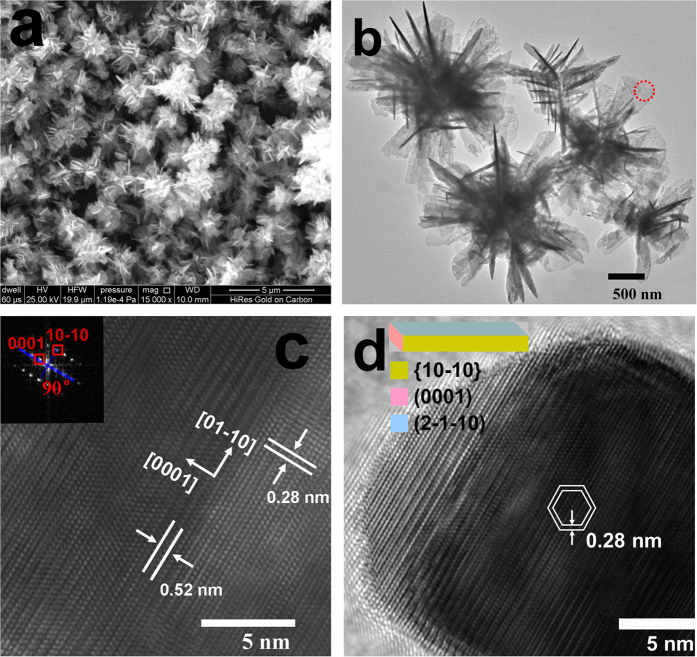
(**a**) SEM image and (**b**) TEM image of S-2; (**c**) HRTEM image of one nanosheet originating from the red circle of b, the inset is the corresponding FFT pattern (viewed along the [2-1-10] axis); (**d**) radial HRTEM image of one nanosheet standing perpendicular to the substrate, the inset is a schematic illustration of the structure of the sheet-like building block.

Figure 2a and b are the SEM and TEM images of S-2, respectively. It can be seen that the sizes of the microflowers are in the range of 1.5–3.0 μm and they are composed of nanosheets with an average thickness of *ca.* 25 nm. HRTEM image (Fig. 2c) taken at the marked area (red circle in Fig. 2b) reveals the highly crystalline nature of the nanosheet. The interplanar spacings are 0.28 and 0.52 nm, corresponding to the interspacing of the (10-10) and (0001) planes, respectively. In addition, the angel labeled in the corresponding FFT pattern is 90° (inset of Fig. 2c), which is identical to the theoretical value for the angle between the (10-10) and (0001) facets. These observations identify the sheet-like building blocks are mainly exposed with (2-1-10) surfaces^20^. As shown in the radial HRTEM of a typical nanosheet (Fig. 2d), similar to the literatures^21^,^22^, the {10-10} atomic planes with lattice spacing of 0.28 nm can be obviously observed; therefore, the nanosheet is surrounded by the {10-10} planes. Based on the above results, as displayed in the inset of Fig. 2d, the mainly exposed facet of ZnO nanosheet is (2-1-10), the side facets are {10-10}, and the top surface is (0001).

**Figure 3:**
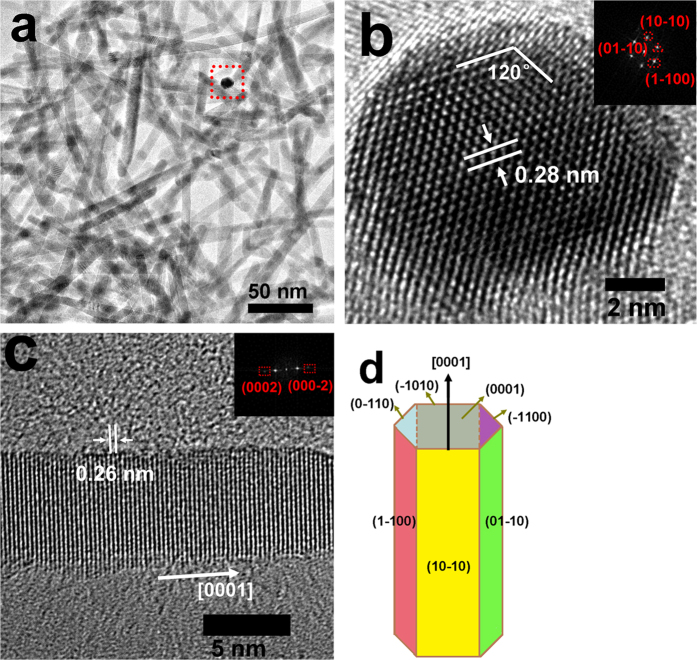
(**a**) TEM image of S-3; (**b**) radial HRTEM image of one nanorod indicated by the red square in a, the inset is the corresponding FFT pattern, which takes along the [0001] zone axis; (**c**) axial HRTEM image of an isolated nanorod, the inset is the corresponding FFT pattern; (**d**) a schematic model of the ZnO nanorod.

As shown in SEM image (Fig. S1b, Supplementary Information), the product (S-3) prepared in pure ethanol medium is entirely composed of disperse ZnO nanorods. The corresponding TEM image (Fig. 3a) indicates that the nanorods have an average diameter of 10 nm and the cross-section of the nanorod is hexagonal shown in the red square. Figure 3b exhibits the radial HRTEM image of a typical nanorod along the *c*-axis with the lattice fringe of 0.28 nm, corresponding to the distance between the {10-10} planes. Figure 3c shows that the long-edge of the ZnO nanorod has a lattice fringe of 0.26 nm, consistent with the interspacing of the (0002) planes, which indicates that the preferred growth direction of the ZnO nanorods is [0001] (*c*-axis). Based on the above results, as illustrated in the schematic model of the ZnO nanorod (Fig. 3d), the hexagonal nanorods are exposed with three kinds of facets, namely {10-10} facets on the column, and (0001) facet on the top, and (000-1) facet on the bottom. Additionally, it should be mentioned that, compared with the primary nanocrystals of S-1, S-3 has a stronger tendency to grow along the *c*-axis and accordingly has longer axial length, thus exposing a larger ratio of {10-10} facets and a smaller ratio of {0001} planes.

**Figure 4:**
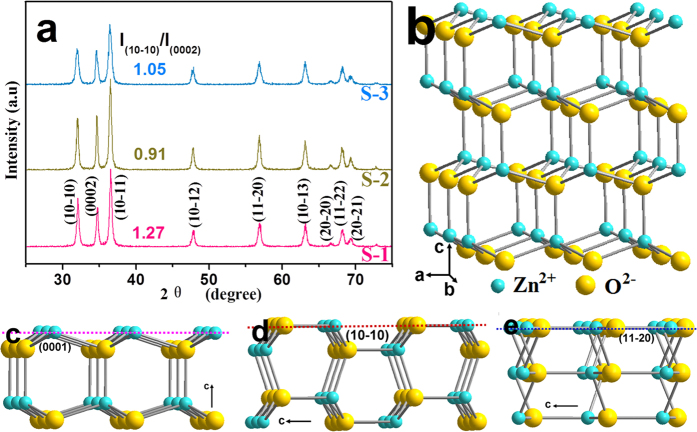
(**a**) XRD patterns of S-1, S-2, and S-3; (**b**) crystal structure of wurtize ZnO; (**c**–**e**) side views of (0001), (10-10), and (2-1-10) facets of wurtize ZnO.

The XRD patterns of S-1, S-2, and S-3 are shown in Fig. 4a. The position of all diffraction peaks match well with the standard XRD pattern of wurtize ZnO (JCPDS No. 36–1451). No peaks for impurities (e.g., Zn(OH)_2_) can be detected, revealing the high purity of the samples. Moreover, the strong diffraction peaks indicate the high crystalline nature of the products. The relative intensity of (10-10) to (0002) (*I*_(10-10)_/*I*_(0002)_) reflects the preferential crystallographic orientation of the samples, and a higher *I*_(10-10)_/*I*_(0002)_ ratio means a larger fraction of polar {0001} planes on the sample surface^16^,^21^. The *I*_(10-10)_/*I*_(0002)_ ratios of S-1, S-2, and S-3 are calculated to be about 1.27, 0.91, and 1.05, respectively, indicating the ratio of exposed {0001} facets decreases from S-1 to S-3 and then becomes minimum for S-2. As an understanding of the structure of ZnO is important to this work, we describe the crystal structure of wurtize ZnO in details. ZnO can be described as hexagonal close packing of Zn and O atoms in P6_3_mc space group, in which Zn atom is tetrahedrally coordinated by O atoms, and vice versa (Fig. 4b); furthermore, ZnO is constructed by a number of positively charged Zn-terminated (0001) facets, alternating with negatively charged O-terminated (000-1) facets, stacked along the *c*-axis. Figure 4c–e are the atomic models of (0001), (10-10), and (2-1-10), respectively. The (0001) plane is metastable and possesses high surface energy, whereas the nonpolar (10-10) and (2-1-10) planes parallel to the *c*-axis are stable^23^.

The BET measurements are performed on S-1, S-2, and S-3, and their specific surface areas are 16.258, 16.688, and 31.454 m^2^ g^−1^, respectively. XPS analysis is performed to investigate the chemical states of Zn and O species in S-1, S-2, and S-3. As displayed in the XPS spectra of Zn 2p (Fig. S2a, c, and e, Supplementary Information), the peaks at 1021.4 and 1044.5 eV are ascribed to Zn 2p_3/2_ and Zn 2p_1/2_, respectively, indicating that Zn exists mainly in the oxidized state Zn^2+^ on the samples surfaces^24^. The O 1 s XPS spectra of S-1, S-2, and S-3 are displayed in Fig. S2b, d, and f (Supplementary Information), and each of them can be fitted into two peaks centered at ~530.2 eV (low binding energy oxygen, O_LBE_) and 531.8 eV (high binding energy oxygen, O_HBE_), respectively. The O_LBE_ could be ascribed to the O atoms coordinating with Zn atoms in the ZnO crystal, whereas the O_HBE_ is caused by the chemisorbed O_2_ in the oxygen deficient regions of ZnO^25^. The terminate structure of ZnO (0001) only contains Zn^2+^ (Fig. 4c); thereby, compared with S-2, S-3 with larger surface area and larger ratio of (0001) planes can adsorb O_2_ more easily and accordingly exhibit O_HBE_ with larger intensity^26^. S-1 has smaller surface area but higher O_HBE_ in comparison with S-2, which is ascribed to the larger percentage of {0001} planes.

### Formation mechanisms of S-1, S-2, and S-3

In the experiment, Zn^2+^ ions are firstly reacted with excessive OH^−^ ions (OH^−^:Zn^2+^ = 4:1) to form [Zn(OH)_4_]^2−^ (Zn^2+^ + 4OH^−^ → Zn(OH)_4_^2−^). Subsequently, with the increasing of the reaction temperature, ZnO nuclei can be formed by the dehydration of [Zn(OH)_4_]^2−^ (Zn(OH)_4_^2−^ → ZnO + H_2_O + 2 OH^−^).

Based on the Gibbs-Wulff’s theory^27^,


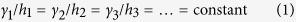


where *γ*_n_ is the surface energy of the facet n and *h*_n_ is the distance between the facet n and a point in the crystal known as Wulff’s point, that is, to minimize the surface energy of a crystal, the crystal planes with smaller surface energy grow slowly and could be exposed on the crystal surface; on the contrary, the fast-growing faces would eventually disappear^28^. As to hexagonal ZnO, the order of surface energy associated with the main crystal planes is *γ*_(0001)_ > *γ*_(10-11)_ > *γ*_(10-10)_, and accordingly the sequence of the growth rates is *ν*_(0001)_ > *ν*_(10-11)_ > *ν*_(10-10)_^29^. As a result, ZnO nuclei usually grow preferentially along the *c*-axis and form into rod-like nanocrystals. Once the original ZnO nanorods generate in the system, to minimize the surface energy, they have a tendency to form aggregates in the growth process and ZnO with diverse shapes can be prepared in different conditions.

**Figure 5:**
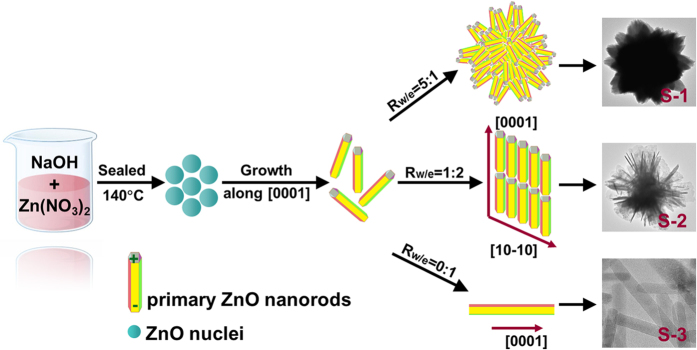
Schematic illustration of the possible formation process of S-1, S-2, and S-3.

Based on the experimental results, it is found that ethanol plays a crucial role on the shapes of the products. We consider that there are two possible reasons. Firstly, according to the classical crystal growth theory, nucleation stage and growth process are involved in the formation of crystal, and these two stages are temporally distinct^30^. The average crystal size can be decreased as the nucleation rate is faster than the growth rate, and vice versa; furthermore, lower surface tension can result in higher nucleation rate, followed by the formation of crystal with smaller size^31^. As to ethanol, its surface tension is about 22 mN m^−1^ against water (73 mN m^−1^). The increase of the ethanol concentration results in a decrease of surface tension, that is, the surface tension of the three different solvents, with *R*_w/e_ value of 5:1, 1:2, and 0:1, is about 40, 30, and 22 mN m^−1^, respectively. The decrease of the surface tension leads to the increase of nucleation rate, so the diameter of the primary ZnO nanorods is decreased stepwise from S-1 to S-2 and S-3. Secondly, H_2_O molecules can attract each other *via* O-H^…^O hydrogen-bonding interaction and form into 3D hydrogen-bonded networks, while hydrogen bonds only occur between different ethanol molecules (C_2_H_5_-O-H^…^OH-C_2_H_5_) and ethanol solvents are 1D and small molecules with short molecular chains. The different spatial structures of H_2_O and ethanol have diverse steric hindrance effects on the agglomeration of the primary nanocrystals, resulting in different final shapes^32^. In the synthetic system, ethanol, served as capping agent, adsorbs on the ZnO surfaces owing to its hydroxyl group^33^,^34^, which can hinder the aggregation of primary ZnO nanocrystals; furthermore, the degree of agglomeration would be reduced with the increase of ethanol. When the R_w/e_ value is 5:1, there is not enough ethanol to adsorb on the ZnO surfaces, so the building blocks have a tendency to aggregate with each other and grow into microspheres (S-1). Furthermore, the aggregation inhibits the *c*-axis oriented growth of the primary nanorods, so they are short in length. As the R_w/e_ value is decreased to 1:2, the original ZnO nanorods are assembled along the *c*-axis driven by the static electrical attraction force owing to the different charge distributions on the {0001} facets; moreover, these primary nanorods also stack along the [10-10] direction for the crystal plane coupling^35^. As a result, the ZnO nanorods are assembled into nanosheets with a high proportion of (2-1-10) plane. Subsequently, these nanosheets are interlaced and overlapped with each other and formed into microflowers (S-2)^20^,^22^. When the solvent is exclusively ethanol (R_w/e_ = 0:1), the nanocrystals have no tendency to aggregate and lead to the formation of isolated nanorods (S-3). As above, the possible formation mechanisms of S-1, S-2, and S-3 are illustrated in Fig. 5.

### Optical properties of S-1, S-2, and S-3

The optical properties of S-1, S-2, and S-3 are investigated by UV-vis diffuse reflectance spectroscopy, and the results are shown in Fig. S3a (Supplementary Information). All spectra present broad intense absorption at wavelength lower than 400 nm, which can be ascribed to the intrinsic bandgap absorption of ZnO for the electron transition from the valence band to the conduction band (O_2p_ → Zn_3d_)^36^. In addition, the light absorption edges of S-3 are blue-shifted from those of S-1 and S-2, which is caused by the strengthening quantum confinement effects of the samples with decreasing their sizes^37^. As to ZnO, a typical direct band gap semiconductor, the band gap energy (*E*_g_) can be calculated from Tauc’s equation which indicates the relationship between the absorption coefficient (*α*) and the photo energy (*hυ*) as follows^38^,


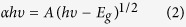


where *h* is Planck’s constant, *υ* is the radiation frequency, and *A* is a constant related to the material and the matrix element of the transition. *α* is obtained by the relation *α* = ln(1/*T*), where the transmittance (*T*) is calculated from the absorbance using Beer-Lambert law. If *αhυ *→ 0, *hυ* = *E*_g_, so extrapolation of the straight line portion to zero absorption coefficient (*α* = 0) in the (*αhυ*)^2^ *vs. (hυ*) plots can give the values of *E*_g_. These plots are given in Fig. S3b (Supplementary Information). It can be seen that the *E*_g_ value of S-2 is 3.14 eV, which is lower than those of S-1 and S-3, namely 3.19 and 3.25 eV, respectively.

### Photocatalytic activities of S-1, S-2, and S-3

**Figure 6:**
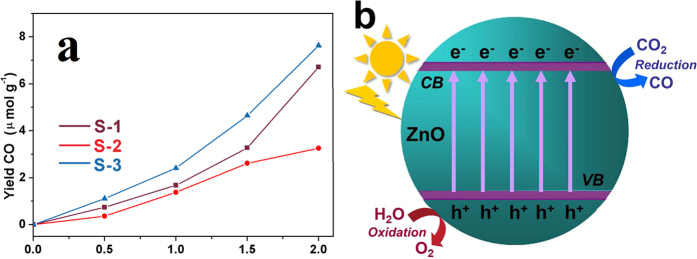
(**a**) CO yield of photocatalytic reduction of CO_2_ over S-1, S-2, and S-3 under sunlight irradiation for 2 h; (**b**) schematic diagram of CO_2_ photoreduction mechanism of ZnO.

The photocatalytic activities of the obtained ZnO catalysts are tested for CO_2_ photoreduction with H_2_O vapor. A series of control experiments indicate that no C1-C2 energy compounds can be generated in the absence of either sunlight irradiation or photocatalyst, illustrating that irradiation and photocatalyst are necessary for the photoreduction of CO_2_. It is found that CO_2_ can be reduced into CO in the present of H_2_O vapor by using S-1, S-2, and S-3. Figure 6a shows the CO production amount from the photocatalytic system containing different ZnO catalysts under sunlight irradiation for 2 h. It can be seen that S-2 shows the lowest photocatalytic activity among the three catalysts, with a CO formation rate of 1.627 μmol g^−1^ h^−1^; S-1 and S-3 exhibit relatively high activity, which are 3.357 and 3.814 μmol g^−1^ h^−1^, respectively. The mechanism of the photoreduction of CO_2_ into CO over the ZnO photocatalysts can be schematically depicted in Fig. 6b. That is, ZnO is irradiated by sunlight and the electrons (e^−^) in the valence band (VB) are excited to the CB with the generation of holes (h^+^) (ZnO + *hυ* → ZnO (e^−^ + h^+^)). Then, the holes (h^+^) lead to the oxidation of water to produce O_2_ (H_2_O + 2 h^+^ → 1/2O_2_ + 2 H^+^ + 2e^−^), while the photogenerated electrons can reduce CO_2_ into CO (CO_2_ + 2 H^+^ + 2e^−^ → CO + H_2_O).

## Discussion

**Figure 7:**
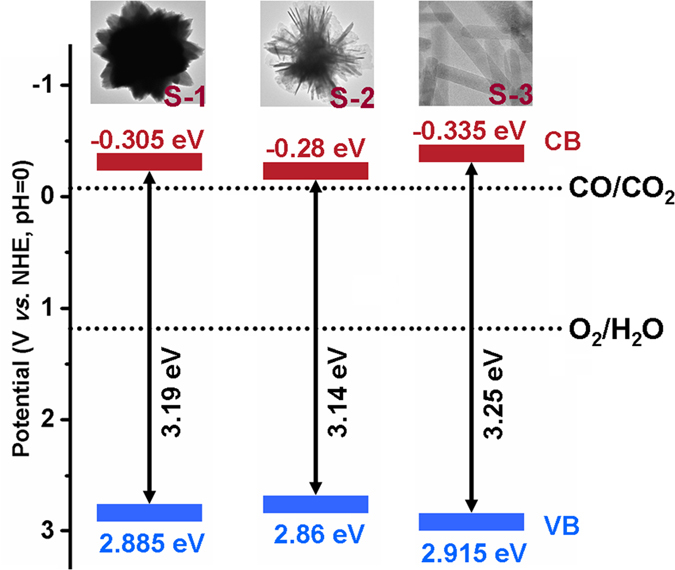
The determined CB and VB potentials of three kinds of ZnO *vs.* NHE (pH = 0).

According to the above results, the comparisons among S-1, S-2, and S-3 are listed in Table 1, indicating that the CO formation rate of the three samples is in the order of S-3 > S-1 > S-2. The different photocatalytic activities of the samples with different ratios of {0001} facets may arise from the following reasons. Firstly, the electronic structure of semiconductor plays an important role in the level of CB and VB, influencing the photocatalytic performance. It is known that the nanocrystals with diverse exposed facets have different *E*_CB_ and *E*_VB_^16^,^39^. As to ZnO, the *E*_CB_ and *E*_VB_ can be calculated by the following empirical equations^40^,^41^:


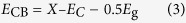



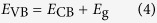


where *X* is the electronegativity of a semiconductor (*X* value for ZnO is ~5.79 eV), *E*_C_ is the energy of free electron on the hydrogen scale (~4.5 eV), and *E*_g_ is the band gap energy of the semiconductor, which can be calculated from the absorption spectra. Thus, the calculated *E*_CB_ of S-1, S-2, and S-3 are −0.305, −0.28, and −0.335 eV, respectively; moreover, the VB values of S-1, S-2, and S-3 are 2.885, 2.86, and 2.915 eV, respectively. On the basis of the above CB and VB values, the relative band positions of the three kinds of ZnO *versus* a normal hydrogen electrode (NHE, pH = 0) are shown in Fig. 7. It can be seen that the redox potentials of the VB holes are sufficiently positive for holes to act as electron acceptors; moreover, the CB values of the three samples are all more negative than the redox potential of CO/CO_2_ (−0.07 eV *vs.* NHE at pH = 0), so the reduction of CO_2_ to CO is thermodynamically possible^42^. Clearly, S-1 and S-3 with larger ratio of {0001}-exposed facets exhibit lower *E*_VB_ and higher *E*_CB_ in comparison with S-2, thus producing more oxidative holes and more reductive electrons^16^. Secondly, as discussed above, the three ZnO are terminated with {0001}, {10-10}, or (2-1-10). Among these low-index facets, the {10-10} and (2-1-10) facets are relatively stable owing to their low surface energies (2.3 and 2.5 J m^−2^, respectively). In contrast, the polar {0001} facets have high surface energy (4.0 J m^−2^) and possess significantly enhanced photocatalytic activity^6^,^22^,^23^. Thirdly, the exposed (000-1) facets terminated with a high density of oxygen atoms favor the formation of oxygen vacancies in the crystal lattice^43^. The oxygen vacancies enhance the efficient separation of electron-hole pairs and improve the trapping capability for CO_2_, thus improving the photoreduction activity of CO_2_^44^,^45^; moreover, oxygen vacancies are more beneficial to the selective photoreduction of CO_2_ into CO than CH_4_^27^,^46^. In these regards, compared to S-2 and S-3, S-1 with more {0001} facets exhibits a higher photocatalytic activity, which can be further clarified by the CO_2_ reduction activity of S-1, S-2, and S-3 (0.2065, 0.0975, and 0.1212 μmol m^−2^ h^−1^, listed in Table 1).

On the other hand, the photocatalytic CO_2_ reduction activity is not only associated with the exposed facets, but also closely related to the CO_2_ adsorption capability on the surface of the photocatalyst^11^. The photocatalyst with large surface area can provide more active sites and reaction centers for the photoreduction of CO_2_, thus exhibiting a higher photocatalytic activity. The surface area of S-3 is about two times larger than that of S-1, which is beneficial for the photocatalytic reaction; therefore, it is not surprising that S-3 has better photoreduction activity than S-1.

In summary, a series of ZnO micro/nanomaterials with different morphologies and exposed facets have been prepared by a solvothermal method. The possible growth mechanisms of these ZnO samples have been proposed. Ethanol, adsorbing on the surfaces of ZnO for its hydroxyl group, has played an important role in the shapes of the products as capping agent. In addition, it was demonstrated that the ZnO nanorods had superior CO_2_ photoreduction performance, ascribed to the synergistic effect of their large ratio of exposed {0001} facets and large surface area. This work may provide some important implications in developing more efficient photocatalysts based on the electronic structure effects induced by the crystal facets.

## Methods

### Chemicals

All chemicals are purchased from Comio Chemical Reagent Co., Ltd. (Tianjin, China), and they are analytical reagent grade and used without further purification.

### Synthesis of S-1, S-2, and S-3

In the synthesis, Zn(NO_3_)_2_·6H_2_O (0.5 mmol) and NaOH (2.0 mmol) are dissolved into 15 mL distilled water-anhydrous ethanol solvent (with a *R*_w/e_ value of 5:1). Subsequently, the mixture is stirred for 20 min, transferred into a Teflon-lined stainless steel autoclave (30 mL), and maintained at 140 °C for 12 h. After the autoclave is cooled to room temperature, the precipitate is collected by centrifugation, washed with distilled water and ethanol, and finally dried in a vacuum oven at 60 °C for 8 h. The same procedure as for the synthesis of S-1 is used for the fabrication of S-2 and S-3, except that the *R*_w/e_ value is decreased to 1:2 and 0:1, respectively.

### Characterizations

The crystal structures of the products are characterized by X-ray diffraction (Rigaku D/max 2500 V/PC) using Cu Kα radiation (λ = 1.54056 Å) in the 2*θ* range from 20° to 80° at a scan rate of 0.05 ° s^−1^. The morphologies of the products are examined with a JSM 6700 F scanning electron microscopy (SEM). Transmission electron microscopy (TEM) and high-resolution TEM (HRTEM) micrographs are taken with a JEM-2100 transmission electron microscopy. X-ray photoelectron spectroscopy (XPS) analysis is performed on a Thermo ESCALAB 250XI multifunctional imaging electron spectrometer using Al Kα as the excitation source, and the data are calibrated by setting the adventitious C 1 s peak at a fixed value of 284.5 eV. UV-Vis diffuse reflectance spectra (DRS) of the samples in the wavelength range of 300–700 cm^−1^ are recorded on a UV-vis spectrophotometer (Hitachi U-3010), using BaSO_4_ as the background. The special surface areas of the samples are performed through measuring the N_2_ adsorption-desorption isotherms at 77 K on an automated adsorption apparatus (Micromeritics 3Flex), using the Brunauer-Emmett-Teller (BET) theory.

### Photocatalytic reduction of CO_2_

The photocatalytic reduction of CO_2_ over the as-prepared ZnO micro/nanomaterials is conducted in Labsolar-IIIAG closed gas system. NaHCO_3_ (1.712 g) is added into the system, and ZnO (0.15 g) is uniformly dispersed on a watch-glass with an area of 28.26 cm^2^ before putting into the reaction cell (Pyrex glass) with a total volume of 350 mL. Then, the above system is vacuum-treated to completely remove the air. Subsequently, 5 mL H_2_SO_4_ solution (4 M) is injected into the reactor to react with NaHCO_3_ to generate H_2_O vapor and 1 atm CO_2_. After that, the reactor is irradiated by a high pressure Xe lamp (300 W) and the photoreaction temperature is maintained at 20 °C in the DC-0506 low-temperature thermostat bath. At each time interval (0.5 h), gas is taken from the reaction cell and then measured by using the GC9790II gas chromatography equipped with a flame ionization detector (GDX-502 columns).

## Additional Information

**How to cite this article**: Liu, X. *et al*. Photocatalytic Reduction of CO_2_ by ZnO Micro/nanomaterials with Different Morphologies and Ratios of {0001} Facets. *Sci. Rep.* **6**, 38474; doi: 10.1038/srep38474 (2016).

**Publisher's note:** Springer Nature remains neutral with regard to jurisdictional claims in published maps and institutional affiliations.

## Supplementary Material

Supplementary Information

## Figures and Tables

**Table 1:** Photocatalytic performance of S-1, S-2, and S-3 for CO_2_ reduction.

Samples	*I*_(10-10)_/*I*_(0002)_	surface areas (m^2^ g^−1^)	*E*_g_ (eV)	CO formation rate (μmol g^−1^ h^−1^)	CO_2_ reduction activity (μmol m^−2^ h^−1^)
S-1	1.27	16.258	3.19	3.357	0.2065
S-2	0.91	16.688	3.14	1.627	0.0975
S-3	1.05	31.454	3.25	3.814	0.1212
