# Supplementary material for: Photocatalytic Reduction of CO2 by ZnO Micro/nanomaterials with Different Morphologies and Ratios of {0001} Facets
Source: Sci Rep. 2016 Dec 6;6:38474. doi: 10.1038/srep38474 (PMC5138834; doi:10.1038/srep38474)
Supplement: Supplementary Information [file srep38474-s1.doc]

**Supplementary Information**

**Photocatalytic Reduction of CO_2_ by ZnO Micro/nanomaterials with Different Morphologies and Ratios of {0001} Facets**

Xiaodi Liu^1^, Liqun Ye^1^, Shanshan Liu^1^, Yinping Li^1^, and Xiaoxu Ji^2^,*

^1^ College of Chemistry and Pharmaceutical Engineering, Nanyang Normal University, Nanyang, Henan, 473200, P. R. China

^2^ College of Physics and Electronic Engineering, Nanyang Normal University, Nanyang, Henan, 473200, P. R. China

^*^ Corresponding author: E-mail: xxji2010@163.com.


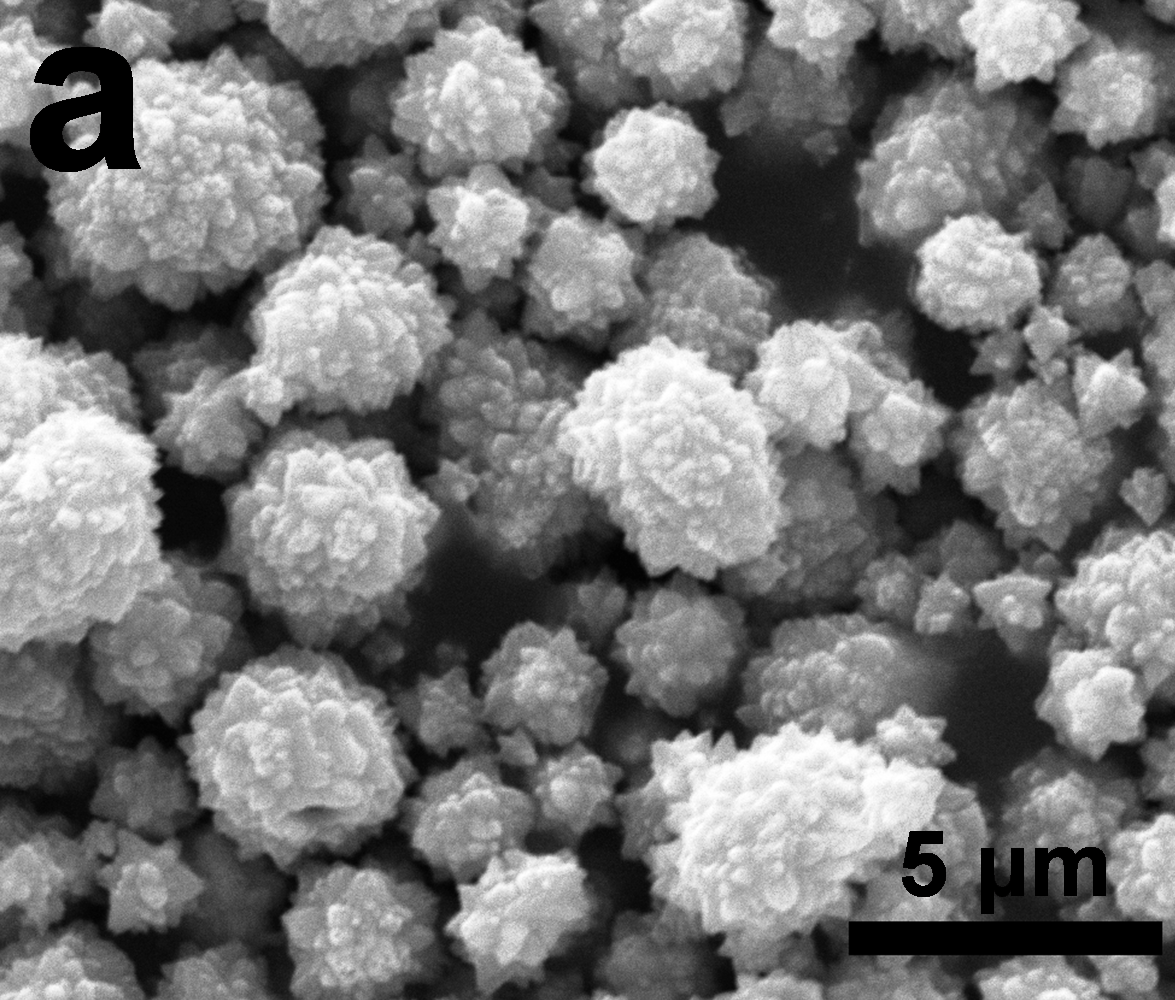

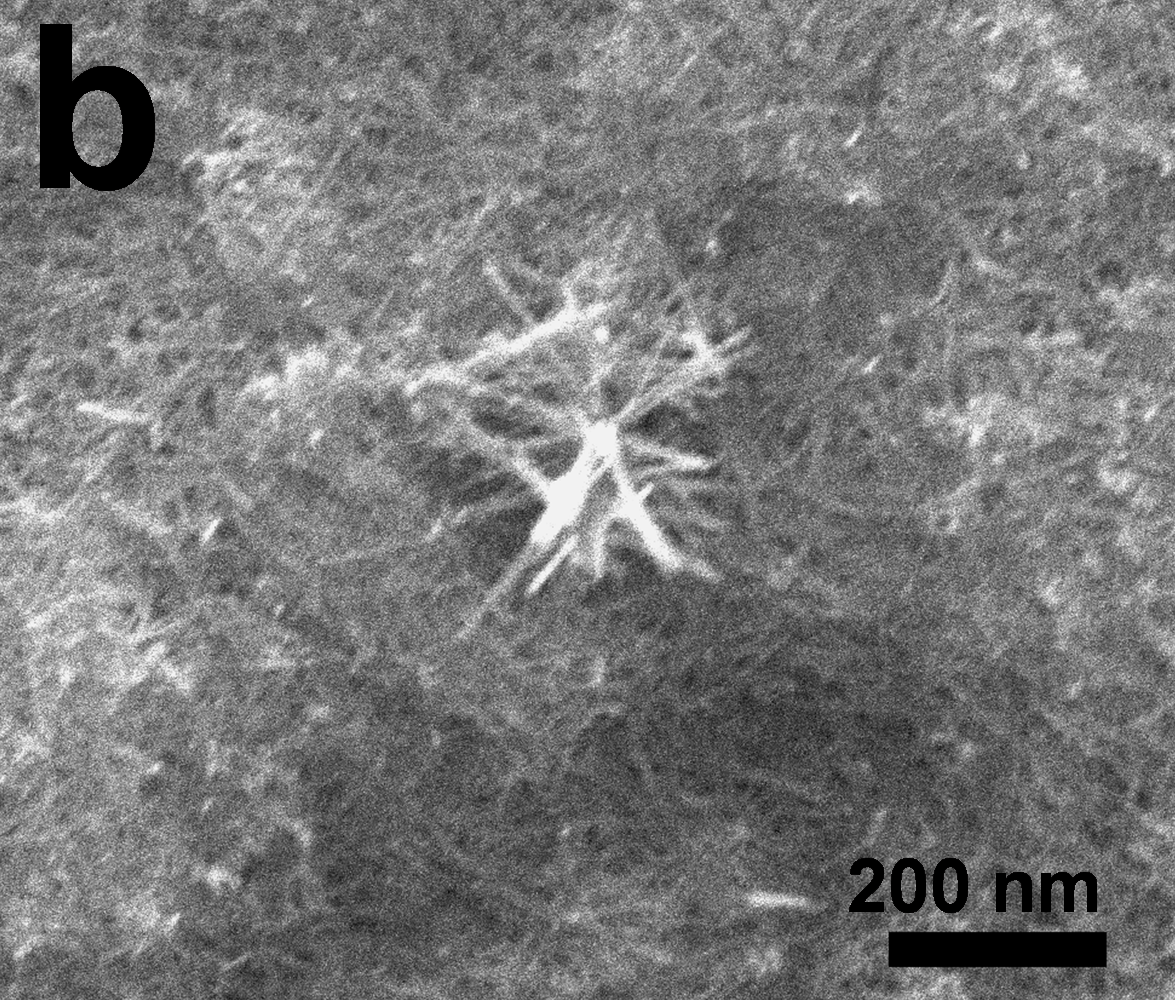


**Figure S1** SEM images of (a) S-1 and (b) S-3.

**Figure S2** Zn 2p and O 1s XPS spectra of S-1 (a and b), S-2 (c and d), and S-3 (e and f).

**Figure S3** (a) UV-Vis diffuse reflectance spectra (DRS) and (b) the (αhυ)^2^ versus photon energy (hυ) plots of S-1, S-2, and S-3.
